# Rivastigmine-Loaded L-Lactide-Depsipeptide Polymeric Nanoparticles: Decisive Formulation Variable Optimization

**DOI:** 10.3797/scipharm.1211-20

**Published:** 2013-03-28

**Authors:** Kunal Pagar, Pradeep Vavia

**Affiliations:** Department of Pharmaceutical Sciences and Technology, Institute of Chemical Technology, University under Section 3 of UGC Act-1956, Elite Status & Centre of Excellence – Govt. of Maharashtra, TEQIP Phase II Funded, Matunga, Mumbai-400019, India.

**Keywords:** Rivastigmine, L-lactide-depsipeptide copolymer, Nanoparticles, Particle size, Formulation variables

## Abstract

The main aim of the investigation was to explore a novel L-lactide-depsipeptide copolymer for the development of rivastigmine-loaded polymeric nanoparticles. L-lactide-depsipeptide synthesis was based on the ring opening polymerization reaction of L-lactide with the cyclodepsipeptide, cyclo(Glc-Leu), using tin 2-ethyl hexanoate as an initiator. Rivastigmine-loaded nanoparticles were prepared by the single emulsion-solvent evaporation technique. The influence of various critical formulation variables like sonication time, amount of polymer, amount of drug, stabilizer concentration, drug-to-polymer ratio, and organic-to-aqueous phase ratio on particle size and entrapment efficiency was studied. The optimized formulation having a particle size of 142.2 ± 21.3 nm with an entrapment efficiency of 60.72 ± 3.72% was obtained. Increased rivastigmine entrapment within the polymer matrix was obtained with a relatively low organic-to-aqueous phase ratio and high drug-to-polymer ratio. A decrease in the average size of the nanoparticles was observed with a decrease in the amount of polymer added and an increase in the sonication time. Prolonged sonication time, however, decreased rivastigmine entrapment. From the different lyoprotectant tested, only trehalose was found to prevent nanoparticle aggregation upon application of the freeze-thaw cycle. Drug incorporation into the polymeric matrix was confirmed by the DSC and XRD study. The spherical nature of the nanoparticles was confirmed by the SEM study. The *in vitro* drug release study showed the sustained release of more than 90% of the drug up to 72 h. Thus, L-lactide-depsipeptide can be used as an efficient carrier for the nanoparticle preparation of rivastigmine.

## Introduction

Rivastigmine tartrate (RT) is a carbamate derivative that reversibly inhibits the metabolism of aceylcholinesterase (AChE) and butyrylcholinesterase (BuChE), preferentially in the central nervous system [1]. RT is chemically 3-[(1*S*)-1-(dimethylamino)ethyl]phenyl ethyl(methyl)carbamate tartrate [2]. It is widely prescribed to patients suffering from mild-to-moderate Alzheimer’s disease. RT has been shown to improve or maintain patients’ performance in three major domains: cognitive function, global function, and behaviour [3]. It is currently marketed as oral solution, capsules, and a transdermal patch. The existing formulations require daily dosing of rivastigmine [4]. However, limitations with its oral therapy include restricted entry into the brain due to its hydrophilicity, necessitating frequent dosing and cholinergic side effects like severe bradycardia, nausea, dyspepsia, vomiting, and anorexia [5, 6]. Hence, the present investigation was aimed at formulating nanoparticulate systems of RT that can improve therapeutic efficacy, provide sustained release, reduce dosing frequency, and minimize side effects.

In recent years, nanotechnology has emerged as a promising platform for drug delivery since it offers a suitable means of delivering small as well as high molecular weight drugs, proteins, peptides, or genes to cells and tissues. Low-dose drugs were found to be successfully encapsulated and effectively delivered using a nanoparticle drug delivery system. Optimization of existing techniques in combination with new methods using biodegradable polymeric carriers enabled them to emerge as suitable delivery systems with increased acceptance and potential [7, 8]. The nanoparticle drug delivery system offers an advantage to cells or tissues due to specific delivery, bioavailability improvement of drugs by increasing their diffusion through biological membranes, and/or to protection of the drug against enzyme inactivation [9, 10]. The nanoparticle drug delivery system involves either conjugation of the drug to the surface of the nanoparticle or encapsulation and protection inside the core. In addition, the delivery systems can be designed to provide either controlled release or a triggered release of the therapeutic molecule [11].

The biodegradable polymeric nanoparticles may be defined as matrix type, solid colloidal particles in which drugs are dissolved, entrapped, encapsulated, chemically bound, or adsorbed to the constituent polymer matrix [12–14]. Nanoparticles have been widely studied in biomedical and biotechnological applications, particularly in drug delivery systems for drug targeting since their particle size (ranging from 10 to 1000 nm) is acceptable for intravenous injection [15–17]. In comparison with other colloidal carriers, polymeric nanoparticles possess both a higher stability in biological fluids and against enzymatic metabolism [18]. The higher stability of the nanoparticles is attributed to the reduced interactions and exchanges with blood components. In addition, the advantage of nanoparticles over other colloidal carriers such as liposomes arises from the possibility of controlling drug release depending on the polymer used [19].

The research reveals that drug uptake into the brain from polymeric NPs is increased which is mainly because of passive uptake of the long circulating NP formulation. In the present research work, it was hypothesized that the hydrophilicity anchored into the polymer due to inclusion of the cyclo(Glc-Leu) group might be imparting the stealth properties of the NP system. This might be the reason for the long circulation duration of drug NPs and passive targeting to the brain by administering the formulation through the intravenous route.

In the present research work, rivastigmine-loaded polymeric nanoparticles were prepared using the single emulsion-solvent evaporation method. The newer polymer L-lactide-depsipeptide was explored as the drug delivery carrier for the nanoparticle preparation whose synthesis was previously reported by our research group [20]. Biocompatibility and biodegradability of the polymer was very well-reported in the literature [20–22]. In this investigation, we studied the effects of excipients and process parameters on the particle size and entrapment efficiency during the preparation of rivastigmine-loaded L-lactide-depsipeptide polymeric nanoparticles.

## Materials and Methods

Rivastigmine tartarate (RT) was procured from Dr. Reddy’s Laboratory (Hyderabad, India). L-lactide obtained from Alfa Aesar (Hyderabad, India) was recrystallized twice before using. Dry tetrahydrofuran (THF) purchased from Merck (Mumbai, India) was used as a polymerization solvent without purification. Tin 2-ethylhexanoate and Polyvinyl alcohol (Mw~31,000) were purchased from Sigma Aldrich (Mumbai, India). The other reagents were of chemical grade and used without further purification.

### Synthesis of the Lactide-depsipeptide copolymer

**Fig. 1:**
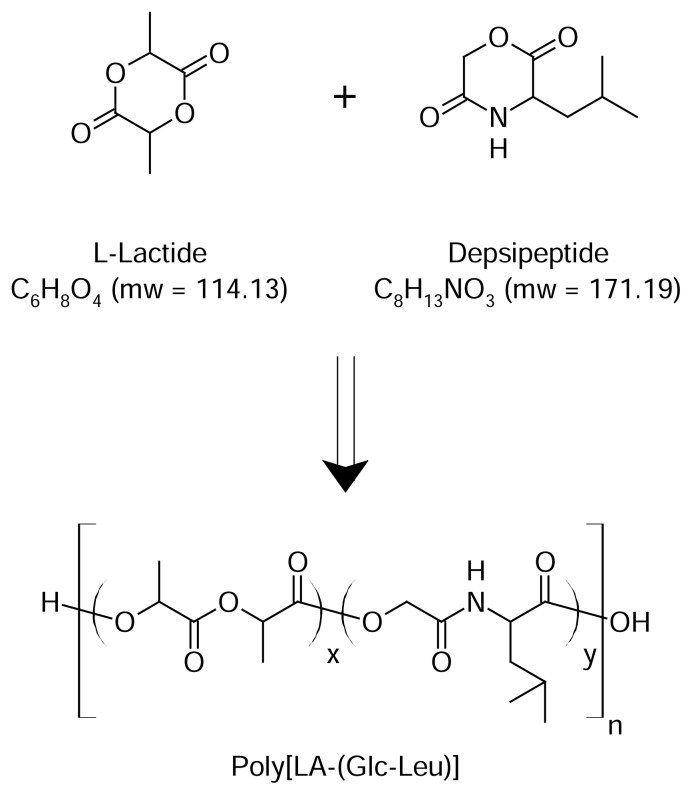
Synthesis of Poly[LA-(Glc-Leu)]

The L-lactide-depsipeptide i.e. poly[La-(Glc-Leu)] copolymer was synthesized by the reaction scheme as shown in Figure 1[20].

Cyclodepsipeptide i.e. cyclo(Glc-Leu) synthesized previously was copolymerized with L-lactide in THF using tin-2-ethyl hexanoate as an initiator by the ring opening polymerization reaction. The purification of the polymer was carried out using the diethyl ether precipitation method in order to remove any traces of monomers and any other impurity [20]. The obtained polymer was analyzed by various analytical techniques. The IR spectra were recorded with the FTIR-5300 spectrophotometer (Jasco, Japan). The ^1^H-NMR spectra were recorded on the Mercury Plus 300 MHz NMR spectrometer (Varian, USA) using tetramethylsilane as the internal reference (Table 1). Gel-permeation chromatography (GPC) analysis was carried out using the Waters GPC System with the Waters2414 RI Detector under the following conditions: Styragel^®^ HT4THF, a 7.8 × 300 mm column, and tetrahydrofuran (THF) were used as the mobile phase at a flow rate of 1.0 ml/min with polystyrene as the standard (Table 2). The Tg and Tm of the polymer were measured by Differential Scanning Calorimeter (DSC) (Perkin Elmer, Pyris-6 DSC, USA) in the range +30 to +250 °C at a heating rate of 10°C/min.

## Experimental

### Preparation of nanoparticles

The nanoparticles were prepared by the oil-in-water (O/W) emulsion solvent evaporation method [6]. Briefly, the polymer (80 mg) and RT (16 mg) were dissolved in methylene chloride (DCM; 2 ml). This organic phase was added slowly under moderate magnetic stirring into double distilled water containing polyvinyl alcohol (0.5% w/v; 10 ml). The resulting mixture was subjected to homogenization using the Ultraturrax (5000 rpm, 30 sec) (T-25 digital Ultraturrax, Ika India Private Ltd., Bangalore, India) in order to get a stable O/W emulsion with diminished droplet size. Further size reduction was done by subjecting the emulsion to sonication using the ultrasonic probe sonicator (Dakshin, Mumbai, India) for 120 s at ultrasonic power inputs (630–650 Amperes). The organic solvent was then removed by evaporation under magnetic stirring for 4–6 h. The entire dispersion was centrifuged at 18,000 rpm for 15 min using a high-speed centrifuge (REMI PR-24, Remi Laboratory Instruments, Mumbai, India) in three cycles. The supernatant was analyzed for its free drug content and the sediment constituting the nanoparticles was freeze-dried. For freeze-drying, pre-freezing of the samples was done at −70°C for 24 h, then the flasks were connected to a freeze-drier (Labconco, USA) under vacuum (1 mbar, −30°C).

The effects of the different formulation variables on particle size and entrapment efficiency were studied. Sonication time and the amount of polymer were varied from 0.5 min to 4.0 min and 80 mg to 400 mg, respectively. The effects of different stabilizer concentrations (0.1, 0.2, 0.5, and 1.0% w/v) and the amount of drug (16 mg, 32 mg, 48 mg, and 80 mg) were also studied. The ratio of the two important excipient variables i.e. drug-to-polymer ratio and organic-to-aqueous phase ratio on drug entrapment and particle size were examined. Only one parameter was changed in each series of experiments.

### Characterization of Nanoparticles

#### Entrapment Efficiency

The drug content in the nanoparticle formulation was determined by analyzing the drug concentration in the supernatant. The formulation was centrifuged at 18000 rpm using a high-speed centrifuge for 15 min and the amount of drug in the supernatant was measured by HPLC with the Jasco Intelligent Unit on Phenomenex, Luna C-18 column (250 × 4.6 mm, Merck, India) at a flow rate of 1.0 ml/min [mobile phase- 0.02M phosphate buffer (pH 3.0):acetonitrile (75:25)] [23].

Eq. 1 $$\text{Entrapment efficiency }(\%)=\frac{\text{Amount of drug intrapped}}{\text{Total amount of drug taken}}\times 100$$

#### Particle size measurement

The size of nanoparticles was determined by Delsa™Nano C (Beckman Coulter, USA) which measured the particle size based on photon correlation spectroscopy. Every sample (0.1 ml) was diluted with deionized water (4 ml) and the reading was carried out at a scattering angle of 165° with respect to the incident beam.

#### Freeze-Thaw study

Selection of the cryoprotectant was done based on the freeze-thaw study. This study was carried out by subjecting the nanoparticle dispersion to freezing for 24 h at −70 °C in a deep freezer (Labtop, Quality lab Equipment, Mumbai, India) followed by thawing at 28 °C. The particle size and PI before freezing and after thawing was determined by PCS.

#### Effect of type and concentration of cryoprotectant

Aliquots of RT nanoparticles having a concentration of 28 mg/ml were taken in vials. Different cryoprotectants like lactose, trehalose, mannitol, sucrose, xylitol etc. were screened during this study. Two different concentrations of the cryoprotectants viz. 2.5% and 5% w/v were evaluated.

#### Freeze-drying and re-dispersibility of nanoparticles

An aliquot of the optimized batch was freeze-dried to verify the physical stability and to check the redispersibility. The nanoparticle suspension was frozen in a deep freezer (Labtop, Quality Lab Equipment, Mumbai, India) at −70 °C and lyophilized using a freeze dryer (Labconco, USA) for 24 h at −30 °C. The freeze-dried product was then evaluated for re-dispersibility in aqueous medium. Precisely 20 mg of the dry product was rehydrated with 10 ml of the phosphate buffer, pH 7.4. The particle size after redispersion and the time taken for the formation of the colloidal dispersion was noted.

### Solid State Characterization of the Optimized Formulation

#### Surface Morphology

A drop of RT-loaded polymeric nanoparticles was placed on double-sided black adhesive tape and fixed onto a graphite surface. The sample was dried at room temperature for an initial period followed by complete removal of the moisture in a desiccator. The stub was sputter-coated with gold and the coated samples were viewed under a scanning electron microscope (Hitachi, Japan) at 5 to 20 kV to reveal the surface morphology of the nanoparticles.

#### Fourier transform infrared (FTIR) spectroscopic analysis

The FTIR spectra of the powder samples were recorded on the FTIR-5300 spectrophotometer (Jasco, Japan) by the KBr disk method using a hydrostatic press to form a compact disc of the samples. The scanning range was 4,000–400 cm^−1^.

#### Differential Scanning Calorimetry

Thermal characterization of the freeze-dried nanoparticles along with the plain drug and polymer were done using a Differential Scanning Calorimeter (Perkin Elmer, Pyris-6 DSC, USA). Alumina was used as the reference material and the analysis was carried out in the nitrogen atmosphere (20 ml/min). All samples were run at a scanning rate of 10 °C/min from 30 °C to 250 °C.

#### Powder X-Ray Diffractometry

The study was carried out using an X-ray diffractometer (Rigaku miniflex, Japan). The radiation was from Ni-filtered CuK, with a wavelength of 1.54 Å having a graphite monochromator. The scanning range was from 0–70° with a scanning rate of 2°/min.

#### In vitro release study

The release of RT from the nanoparticles was studied using the dialysis bag diffusion method [24]. Two millilitres of the nanodispersions (equivalent to 1.5 mg of RT) were placed in the dialysis bag (Himedia, Cut off 12,500 Da), sealed, and suspended in a beaker containing 40 mL of phosphate buffer saline (PBS, pH 7.4). The entire system was kept at 37 ± 2°C with continuous stirring on a mechanical shaking bath (100 cycles/min). Samples were withdrawn from the receptor media at predetermined time intervals and replaced with fresh buffer. The amount of RT in the samples was determined by the HPLC (high-performance liquid chromatography) method.

For HPLC (Jasco, Japan) analysis, a reversed-phase Phenomenex, Luna C-18 column (250 × 4.6mm, Merck, India) was used. The mobile phase was a mixture of phosphate buffer (0.02M, pH 3.0): acetonitrile (75:25v/v). The injection volume was 20μl. The flow rate was adjusted to 1.0ml/min (Jasco PU 980 Intelligent HPLC pump) and the wavelength was set to 210 nm (Jasco PU 980 Intelligent UV/VIS detector). Samples were injected using an autosampler (AS-2055 plus intelligent sampler) after suitable dilution with the mobile phase, and the chromatograms were analyzed using Spectra Manager software (Jasco, Japan) provided with the system.

#### Mechanism of drug release

Model-dependent (curve fitting) methods were used for the evaluation of the drug release data [25]. For the model-dependent analysis, two theoretical models describing drug release from the polymeric systems according to Higuchi and Korsmeyer–Peppas were used. Higuchi describes drug release as a diffusion process based on Fick's law according to the following equation (Eq. 2):

Eq. 2 $$\frac{\text{Mt}}{\text{M}\infty }=\text{k}\sqrt{\text{t}}$$

where k is a constant reflecting the formulation characteristic, and M_t_ and M_∞_ are cumulative amounts of the released drug at time t and infinite time, respectively. According to this model, a straight line is expected for the plot of the amount of drug release versus the square root of time if the drug release from the matrix is based on a diffusion mechanism. The Korsmeyer–Peppas model takes into account that the drug release mechanism often deviates from Fick's law and follows anomalous behavior described by the following equation (Eq. 3):

Eq. 3 $$\frac{\text{Mt}}{\text{M}\infty }={{\text{k}}^{\prime }\text{t}}^{\text{n}}$$

where k′ is the kinetic constant and n is an exponent characterizing the diffusion mechanism. The n value is used to characterize different release mechanisms as given in Table 3 for spherical shaped matrices.

## Results and Discussion

Drug and polymer solubility are the key factors in the selection of the particular method for encapsulation [26]. In our study, the single emulsion-solvent evaporation method was adopted for the preparation of RT-loaded polymeric nanoparticles as both the polymer and drug were found to be soluble in the organic phase. The effects of different formulation variables on the particle size and entrapment efficiency were studied in detail.

### Effect of the amount of polymer

**Fig. 2:**
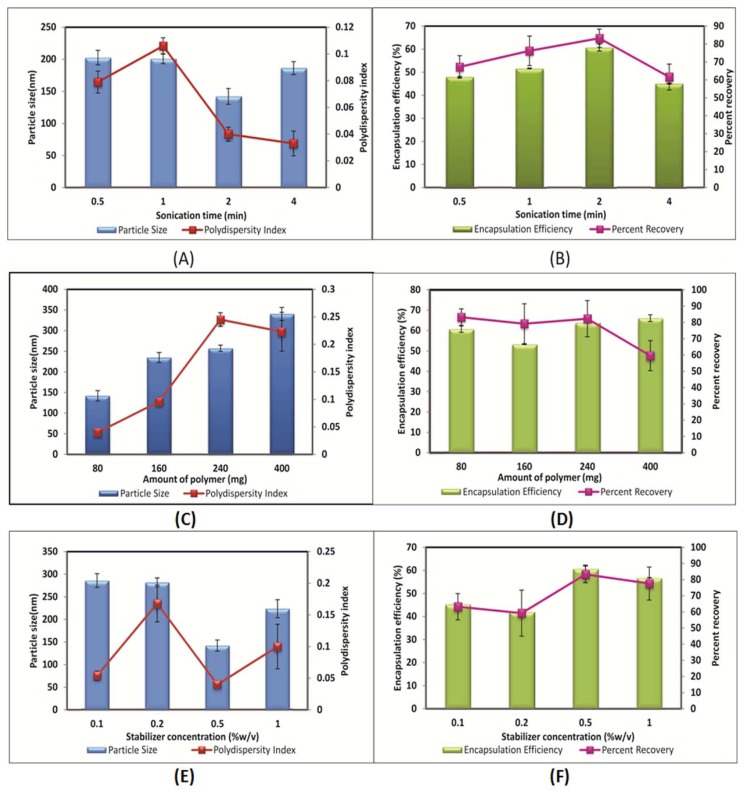
Effect of sonication time on (A) Particle size and polydispersity index (B) Entrapment efficiency and percent recovery; Effect of amount of polymer on (C) Particle size and polydispersity index (D) Entrapment efficiency and percent recovery; Effect of stabilizer concentration on (E) Particle size and polydispersity index (F) Entrapment efficiency and percent recovery

The amount of polymer is one of the decisive formulation variables that plays a crucial role in modulating the particle size and entrapment efficiency of the nanoparticles. Keeping all other parameters constant, the effect of the amount of polymer on the properties of the nanoparticles was investigated. Four different concentrations of the polymer with the same molecular weight were considered (80–400 mg). By increasing the amount of polymer from 80 mg to 400 mg, the particle size and entrapment efficiency were found to increase as depicted in Figure 2(C) and Figure 2(D). This comes from the fact that a viscous polymer solution is more difficult to break up into smaller droplets at the same input power of mixing which led to an increase in particle size. Meanwhile, at a higher amount of polymer, solidification of nanoparticles is more rapid which may lead to the formation of a viscous polymer in the nanodroplets. These results were ascribed to the required shorter time for solidification of the sample with 400 mg of polymer compared to another sample. A higher amount of polymer yields a higher entrapment efficiency for RT, which can be explained by the increased viscosity of the organic phase and denser structure, and results in less drug loss during the evaporation process. These results are in accordance with previous literature [29–31]. An increase in viscosity of the organic phase was a consequence of an increase in the amount of polymer, and this resulted in poor dispersibility of the organic phase into the aqueous phase. Coarse dispersions were obtained at a higher amount of polymer, which lead to the formation of bigger particles during the diffusion process. This can also be due to the insufficient amount of stabilizer present in the aqueous phase for that particular polymer amount.

### Effect of stabilizer concentration

In the present study, an increase in concentration of the stabilizer (polyvinyl alcohol, PVA) from 0.1 to 0.5% w/v made a significant decrease in the size of the nanoparticles prepared with the synthesized polymer. At 1.0% w/v of PVA concentration, there was a slight increase observed in the size of the nanoparticles. This might be because at 1% w/v concentration, the increased viscosity of the aqueous phase might cause a hindrance to the energy input used for the size reduction of the droplets. This resulted in bigger organic phase droplets, which consequently led to increased particle size. Therefore, 0.5% w/v of PVA was considered optimum for the preparation of the nanoparticles. The effect of stabilizer concentration on the mean size of the particles is shown in Figure 2(E). The amount of surfactant plays an important role in the protection of the particles because it can avoid the agglomeration of particles [8, 32]. The polydispersity index values of the formulations prepared are less than 0.2 for all formulations prepared in the study indicating a narrow and homogenous size distribution.

A decrease in drug entrapment was observed with an increase in stabilizer concentration up to 0.2% w/v. The effect of stabilizer on the entrapment efficiency of the formulations is shown in Figure 2(F). An increase in stabilizer concentration up to 0.5% w/v did not influence the entrapment efficiency of RT. However, a slight decrease in the entrapment efficiency was obtained when the stabilizer concentration increased to 1.0% w/v. When the concentration of stabilizer is increased, it helps in solubilizing the drug in the aqueous phase. Due to this, when organic solvent is added to the aqueous phase, a greater amount of RT is soluble in the aqueous phase and assists in drug leakage from the nanoparticles.

### Effect of the amount of drug

**Fig. 3:**
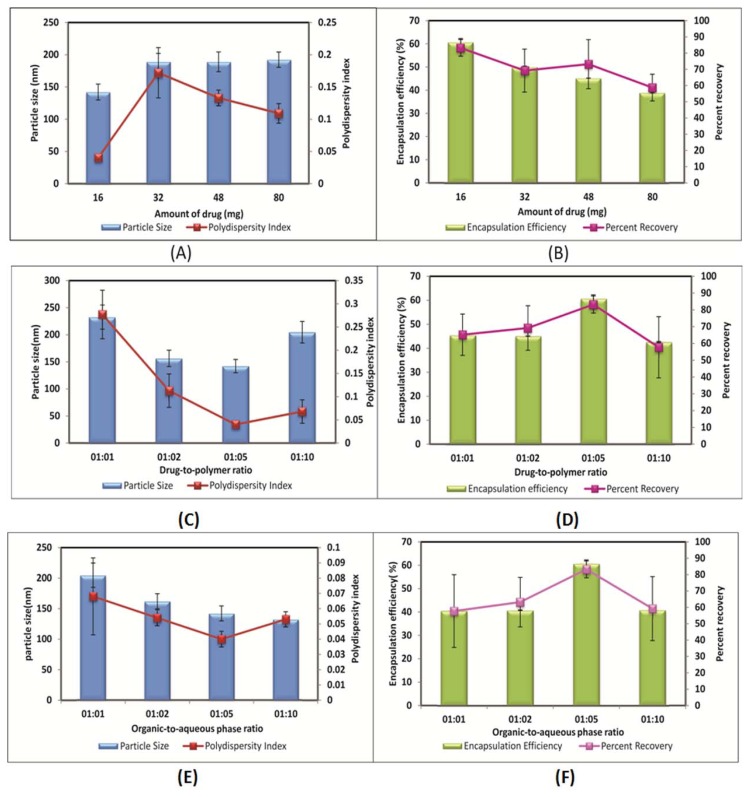
Effect of amount of drug on (A) Particle size and polydispersity index (B) Entrapment efficiency and percent recovery; Effect of drug-to-polymer ratio on (C) Particle size and polydispersity index (D) Entrapment efficiency and percent recovery; Effect of organic-to-aqueous phase ratio on (E) Particle size and polydispersity index (F) Entrapment efficiency and percent recovery

Maintaining a constant initial mass of polymers (80 mg), the mass of RT was varied between 16 and 80 mg. It was observed that the increase in the amount of RT from 16 to 80 mg increased the nanoparticle mean diameter from 142.2 nm to 192.2 nm as shown in Figure 3(A). The reason might be that when a smaller amount of RT was taken initially, smaller particles were produced after the evaporation of the organic solvent, whereas if the amount of RT added initially were increased, the mean particle size would increase because of the high solid content after evaporation. The entrapment efficiency decreased for all batches when the RT amount was increased from 16 to 80 mg as shown in Figure 3(B). This could be due to an inadequate amount of polymer present in the system, being insufficient to entrap the drug inside the matrix.

### Effect of the drug-to-polymer ratio

Keeping the other parameters constant, the drug-to-polymer ratio was varied from higher to lower values i.e. 1:1 to 1:10. With a decrease in the drug-to-polymer ratio, the particle size of the formulation was found to be significantly decreased up to 1:5 (Figure 3C). At a 1:10 drug-to-polymer ratio, it was slightly increased which might be because of an increase in the amount of polymer results in increase in viscosity of organic phase. Increased viscosity might cause hindrance to the size reduction of the organic phase droplets. Thus, a 1:5 drug-to-polymer ratio was found to be optimum as the lowest particle size with the highest drug entrapment of about 60% was obtained at this ratio as shown in Figure 3(D).

### Effect of the organic-to-aqueous phase ratio

The volume of the organic solvent was an important factor and it also controlled the size of the nanoparticles formed [33]. As shown in Figure 3(E), with the increase in volume of the organic phase with respect to the aqueous phase, there was a considerable increase in the size of the nanoparticles. This could be because at the highest organic-to-aqueous phase ratios, coarser emulsions were formed. In addition, a stabilizer concentration at this organic-to-aqueous phase ratio was not sufficient to stabilize the formed emulsions and thus resulted in the formation of emulsions having a higher droplet size. At a 1:5 organic-to-aqueous phase ratio, the highest drug entrapment with minimum particle size was obtained and thus found to be at an optimum ratio for the nanoparticle preparation.

#### Selection of cryoprotectant

**Fig. 4:**
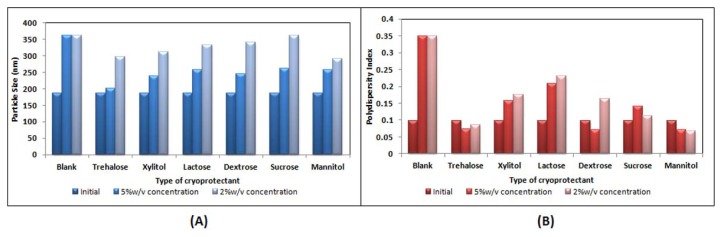
Effect of the Freeze-Thaw cycle in the presence of various types of cryoprotectant on (A) Particle size (B) Polydispersity index

The therapeutic efficiency of the nanoparticles depends upon their physicochemical properties [34]. Degradation of the polymer, drug leakage, drug degradation, and/or drug desorption are the different phenomena occurring when nanoparticles are stored as an aqueous suspension. Long-term stability of the nanoparticles was reported to be successfully achieved using the lyophilisation technique [35, 36]. The freeze-thaw study was reported as a quick and economical method to assess the effect of cryoprotectant on particle aggregation [37]. Figure 4 shows concentration-dependent cryoprotection of the nanoparticles which reveals a greater increase in particle size at lower cryoprotectant concentrations. Nanoparticles frozen without cryoprotectant showed a significant increase in particle size. The polydispersity index was also found to be tremendously increased without cryoprotectant and showed a concentration-dependent increase with respect to cryoprotectant. At equivalent concentrations, trehalose turned out to be a better cryoprotectant than others as it showed a minimum increase in particle size and polydispersity index. Trehalose at 5% w/v revealed a minimal increase in particle size with a Sf/Si ratio (Sf—final size, Si—initial size) of 1.07, where a value less than 1.3 is considered acceptable [38], while other cryoprotectant at the same concentration revealed a Sf/Si ratio of more than 1.3 after the freeze–thaw study. At lower concentrations of trehalose it showed a high Sf/Si as shown in Table 4. Thus, the freeze-thaw study revealed that trehalose was found to be a better cryoprotectant [39, 40].

### Freeze-drying and re-dispersibility of nanoparticles

**Fig. 5:**
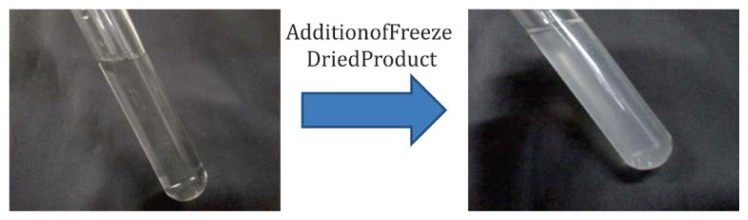
Re-dispersibility of freeze-dried nanoparticles

The correlation between the freeze-thaw study and freeze-drying was studied by freeze-drying nanoparticles at 5% w/v concentration of trehalose. Freeze-dried samples with trehalose showed similar behaviour in particle size as that of the freeze-thaw study. The freeze-dried samples with trehalose showed a bare increase in particle size with a Sf/Si ratio of 1.11. Figure 5 shows the good re-dispersibility behaviour of the freeze-dried nano-particles with trehalose as the cryoprotectant. Thus, for long-term storage of the nanoparticles, lyophilisation is the best method for the conversion of the aqueous solution of the nanoparticles into solid products. The freeze-dried formulation must be reconstituted into physiological solution to be the same as its original aqueous solution immediately before use [41, 42].

#### Solid State Characterization of the Optimized Formulation

Solid state characterization of the freeze-dried RT-loaded polymeric nanoparticles confirmed the internalization of the drug into the polymeric matrix.

### Surface Morphology

**Fig. 6:**
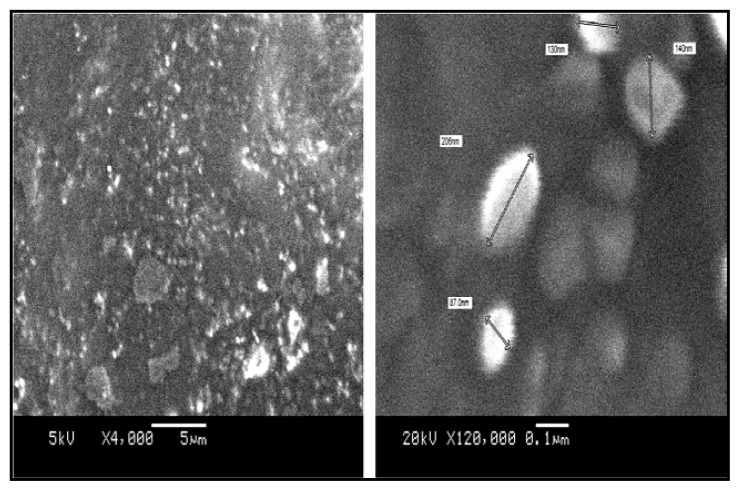
Scanning Electron Microscopy of RT-loaded nanoparticles

Morphological studies of the RT nanoparticles were visualized using scanning electron microscopy (Figure 6). The study affirmed the spherical nature of the particle with mostly uniform size distribution with the particle size between 100–200 nm. The particle size obtained from the SEM study was in agreement with the data obtained from the Delsa™Nano C particle size analyzer.

### Fourier transform infrared (FTIR) spectroscopic analysis

**Fig. 7:**
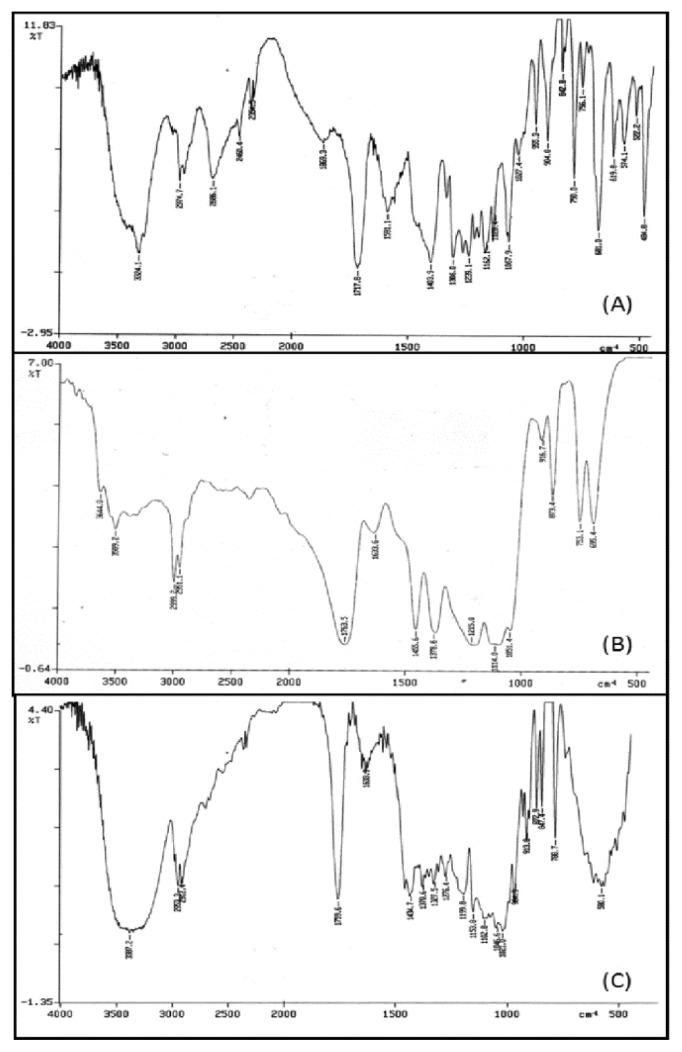
FTIR spectra of (A) RT (B) L-lactide-depsipeptide copolymer (C) RT-loaded polymeric nanoparticles

Fourier transform spectroscopy analysis confirmed the chemical interaction between the drug and the polymer during the nanoparticle formation. The FTIR spectrum of RT showed the characteristic peak of carbamate at 1717.8 cm^−1^ as shown in Figure 7. This peak of RT was found to be merged with the strong CONH stretch of the polymer. This might be because of the dilutional effect of the polymer as that of the drug concentration. C-H stretching in RT appeared at 2974.7 cm^−1^ which appeared to remain the same in the nanoparticulate system indicating no existence of the different association form of RT with the synthesized polymer.

### Differential Scanning Calorimetry

**Fig. 8:**
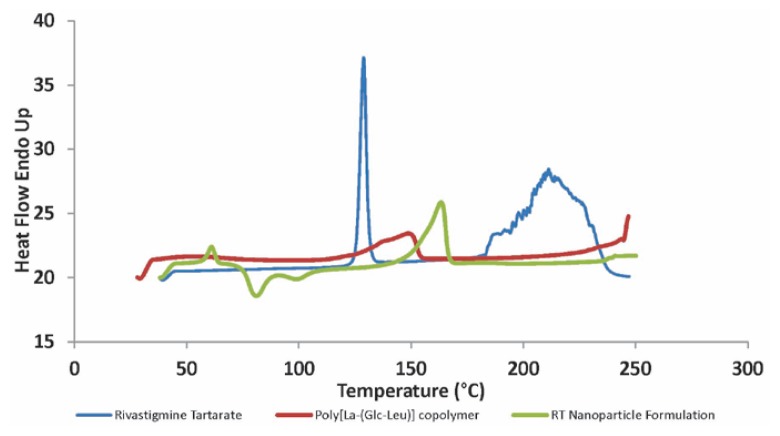
DSC thermograms of Rivastigmine Tartarate (RT), L-lactide-depsipeptide copolymer, and RT-loaded polymeric nanoparticles

Thermograms of RT, synthesized polymer, and RT-loaded nanoparticle formulation are shown in Figure 8. The DSC study reveals the state of the encapsulated drug whether it is dispersed in a microcrystalline form, without polymorphic change, or in transitional change in amorphous form [43]. RT showed a characteristic melting point endotherm near 128.8 °C while the synthesized polymer showed the melting endotherm at 148.8 °C. The freeze-dried RT-loaded nanoparticle formulation showed complete absence of the drug endotherm while the polymer endotherm shifted considerably to the lower side. The absence of the drug endotherm might be due to the complete homogeneous matrix formation of the polymer with the drug or to the dilutional effect of the polymer. This is in agreement with the reported literature [43, 44]. The shifting of the polymer endotherm to the lower side indicated some interaction of the drug with the polymer. The results obtained suggested that the nanoparticles consisted of a homogeneous amorphous drug polymer matrix.

### Powder X-Ray Diffractometry

**Fig. 9:**
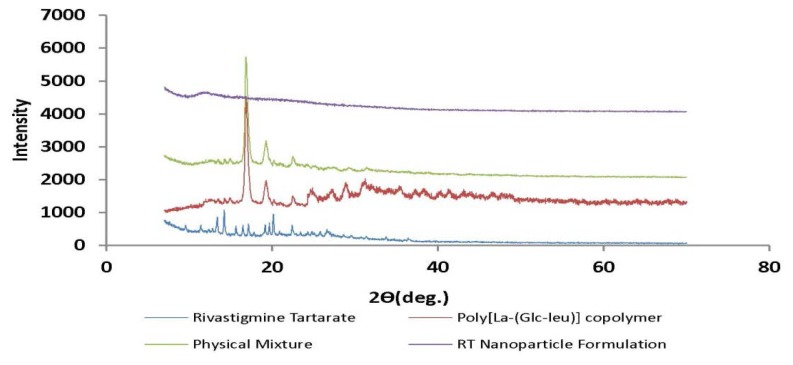
XRD spectra of Rivastigmine Tartarate (RT), L-lactide-depsipeptide copolymer, Physical Mixture, and RT-loaded polymeric nanoparticles

Solid state analysis of the prepared freeze-dried nanoparticulate system by X-ray diffractometry is shown in Figure 9. The XRD data showed that the drug is dispersed in the polymeric matrices in a microcrystalline form, without polymorphic change or transitional phenomena into another form. The XRD spectra of the freeze-dried suspensions showed that by the increased polymeric weight fraction, the intensity of the typical drug peaks were lowered due to the dilutional effect exerted by the polymer network but without a qualitative variation of the drug diffractogram. This decrease in the crystallanity of the drug in formulation affirmed the drug amorphisation and subsequent internalization into the polymeric system.

### In vitro release study and mechanism of drug release

**Fig. 10:**
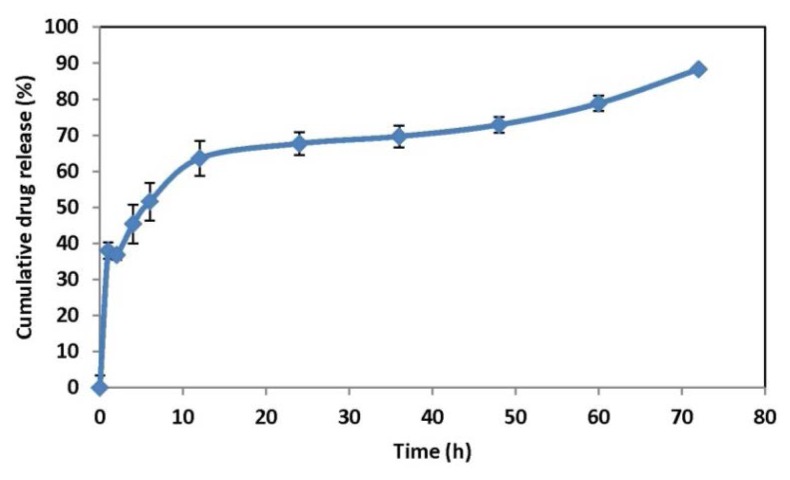
*In vitro* drug release of RT-loaded polymeric nanoparticles in phosphate buffer saline (PBS, pH 7.4)

The *in vitro* dissolution profile of the optimized batch of the RT-loaded poly(lactide depsipeptide) nanoparticles is shown in Figure 10. In this formulation system, the release of RT was found to be complete and the formulation tested showed a biphasic release pattern: one initial fast release followed by a second slow release phase (extended release). The burst effect could be attributed to the escape of the drug from the surface of the polymeric system because the RT present in the polymeric matrix diffuses to the release medium through the pores and channels of the polymeric nanoparticles. The kind of polymer also affected drug release behaviour, as the L-lactide-depsipeptide polymer was found to be more hydrophilic and amorphous; these properties were also responsible for the biphasic release pattern. Furthermore, the homogenous and finer dispersion of the drug molecules in the polymer matrix enhances the dissolution allowing the better penetration of the dissolution medium through the nanoparticles. The dispersion of RT in the polymer matrices led to a gradual dissolution and release of the drug, which was complete within 72 h.

The release data were analyzed on the basis of the zero-order, first-order, and Korsmeyer–Peppas equations and Higuchi kinetics. The release rates, k and n of each model, were calculated by linear regression analysis using the Microsoft Excel Add-Ins DD Solver. The coefficients of correlation (r^2^) were used to evaluate the accuracy of the fit. The r^2^, n, and k values are given in Table 5. Comparative data obtained from the coefficient of regression value (r^2^) indicate that the formulation predominately follows first-order release kinetics which is suggestive of concentration-dependent drug release. Meanwhile, the fitting of the Korsmeyer–Peppas model gave the idea of a diffusion exponent value. The diffusion coefficient value of the optimized formulation (n=0.200) designated that the formulation showed Fickian diffusion kinetics in which diffusional release occurs by the usual molecular diffusion of the drug due to a chemical potential gradient.

## Conclusion

Rivastigmine-loaded nanoparticles were prepared successfully using the single emulsion-solvent evaporation method in the presence of PVA as a stabilizer. This method was found to be simple and produced nanoparticles with a narrow size distribution and good entrapment efficiency. The particle size and drug entrapment were optimized based on the study of the effect of different formulation variables. Use of the newly synthesized L-lactide-depsipeptide copolymer produced RT-loaded nanoparticles with low particle size and high entrapment efficiency of rivastigmine. A change in the concentration of the stabilizer, polymer, and the amount of RT was found to vary the size, polydispersity, and entrapment efficiency of the prepared nanoparticles. The *in vitro* release study revealed the sustained release of RT for 72 h. The effect of different formulation variables helped in the optimization of the formulation in achieving higher efficacy with an improved safety profile of polymer-based nanoparticle formulation. These results justify further investigation of the suitability of these nanoparticles for application in the controlled delivery and/or targeting of RT. Such a formulation approach can be an alternative to improve the stability of the drug with a probable enhancement in absorption, bioavailability, and therapeutic efficacy.

## Figures and Tables

**Tab. 1:** IR, NMR, and DSC data of the L-lactide-depsipeptide copolymer

IR (cm^−1^, KBr disk)	3509 (CONH), 1763 (CO), 1215 (COO)
^1^H-NMR (CDCl_3_)	0.913 (br,CH(CH_3_)_2_; 1.570 (s, CH_3_); 5.133 (s, OCH_2_CO); 5.155(s, CH); 5.2 (s, CHCH_3_)
DSC	Tg = 37.24 °C; Tm =148.81 °C

**Tab. 2:** Molecular weight determination of L-lactide-depsipeptide copolymer

	Yield (%)	M_w_	M_n_	M_w_/M_n_
L-lactide-depsipeptide	92.42	11953	17250	1.44

**Tab. 3:** Diffusion exponent and solute release mechanism for spherical shapes

Diffusion Coefficient(n)	Overall solute diffusion mechanism
n < 0.5	Fickian diffusion
0.5 < n < 0.85	Anomalous (non-Fickian) diffusion
0.85 < n < 1	Case II transport
n > 1	Super Case II transport

**Tab. 4:** Effect of type and concentration of cryoprotectant after the Freeze-Thaw study

Sr. No.	Cryoprotectant	Concentration (%w/v)	Initial size (Si) (nm)	Final size (Sf) (nm)	Sf/Si ratio
1	Blank	–	187.2 ± 5.9	361.1 ± 8.1	1.93
2	Trehalose	2	187.2 ± 5.9	297.7 ± 3.2	1.59
		5	187.2 ± 5.9	201.8 ± 2.12	1.07
3	Xylitol	2	187.2 ± 5.9	312.3 ± 6.6	1.67
		5	187.2 ± 5.9	238.8 ± 8.1	1.27
4	Lactose	2	187.2 ± 5.9	333.4 ± 12.1	1.78
		5	187.2 ± 5.9	257 ± 7.2	1.37
5	Dextrose	2	187.2 ± 5.9	340.5 ± 2.3	1.82
		5	187.2 ± 5.9	244.9 ± 5.1	1.30
6	Sucrose	2	187.2 ± 5.9	361.3 ± 9.7	1.93
		5	187.2 ± 5.9	262 ± 7.2	1.40

**Tab. 5:** Values of r^2^, k, and n for the optimized formulation

Type of Model	r^2^	k	n
Zero Order	0.8218	1.526	–
First Order	0.9262	0.097	
Higuchi	0.9180	11.857	
Kornsmeyer-Peppas	0.9915	35.515	0.200
